# Mr. Reece, on an Instrument for Extracting Teeth

**Published:** 1801-08

**Authors:** R. Reece

**Affiliations:** Surgeon. Craven Street


### *To the Editors of the Medical and Physical Journal.*

GENTLEMEN,

THROUGH the medium of your very ufeful Publication I beg leave to recommend to the attention of the medical world an inftrument for the extraction of teeth, invented by Mr. WHITFORD, Surgeon's Inftrument Maker at St. Bartholomew's Hofpital. Its fuperiority to thofe in common ufe confifts in its elevating the tooth in nearly a perpendicular direction; tion; and from the heel of the inſtrument being applied to the tooth, exactly oppoſite the point of the claw, much injury to the gum and alveolar proceſs, as well as great pain to the patient, is avoided. I have repeatedly made uſe of it with great ſatisfaction to myſelf, and my patients have uniformly expreſſed their ſurprize at the ſlight pain they experienced in the operation. In juſtice to the ingenuity and induſtry of the inventor, I am induced to ſolicit the favour of you to inſert this conciſe account of it in the next number of your Journal.

I am, &c.

*Craven Street,*
*July* 16, 1801.

R. REECE, Surgeon.

